# From glass to life: a commentary on the assessment of the reproductive potential of cryopreserved human oocytes

**DOI:** 10.1007/s10815-022-02565-2

**Published:** 2022-07-16

**Authors:** Carlos E. Plancha, Borut Kovačič

**Affiliations:** 1 Inst. Histologia E Biol. Desenvolvimento, Faculdade de Medicina, Universidade de Lisboa, Lisbon, Portugal; 2 Centro Médico de Assistência à Reprodução – CEMEARE, Lisbon, Portugal; 3 Department of Reproductive Medicine and Gynaecological Endocrinology, University Medical Centre Maribor, Maribor, Slovenia

Oocyte cryopreservation is one of the more recently established methods of assisted reproduction technologies (ART). Vitrification procedures have helped to overcome most technical difficulties enabling better oocyte survival after warming. There are different indications for oocyte cryopreservation. The technology can be used if the partner fails to produce sperm for an IVF treatment on the day of oocyte collection. For patients at risk of ovarian hyperstimulation syndrome (OHSS), we could also opt not only for “freeze-all embryos” but possibly also for “freeze-all oocytes.” Also, women who may have no partner or may lose their fertility potential due to surgery, chemotherapy, radiotherapy, autoimmune diseases, medical conditions causing ovarian insufficiency, or hematopoietic stem cell transplantation could store their oocytes for future use. Planned oocyte cryopreservation can also be used as a tool to help mitigate age-related fertility decline regardless of whether a patient is partnered. All such procedures raise questions both to patients and healthcare professionals: Can we predict the chance for the parenthood from one medically assisted reproduction (MAR) cycle with cryopreserved oocytes? What is the minimal number of cryopreserved oocytes needed to have a reasonable likelihood of success? Is there an upper age limit for women to store their oocytes?

The calculated low probabilities of success and the observed higher risk of pregnancy complications in women aged 40 and above certainly lead to the consideration of setting an upper maternal age limit for both oocyte storage and use. However, many recommendations argue that, for biological reasons, the decision should be made on an individual basis [1]. Most papers analyzing the outcomes of oocyte vitrification focus on cell survival and fertilization efficiency. Recorded data has led to a growing perception, among health professionals that vitrified oocytes behave similarly to fresh oocytes [2]. In patients, this might lead to an overoptimistic belief that oocyte cryopreservation can fully overcome age-related infertility.

Perception of “success in ART” has seen many modification trends. From the initial focus on pregnancy rate to the current live birth rate, a wider perspective is growingly being considered. Higher-order pregnancies are slowly but steadily decreasing, with both patients and health professionals putting the aim on a single healthy birth, particularly in countries implementing a strict eSET policy. All these modifications increased the complexity on effectively evaluating ART success from oocyte cryopreservation cycles.

Since cryopreserving oocytes ultimately aims to achieve a live birth, we should recognize what is the fresh oocyte’s potential to result in a live birth, so that we can build realistic expectations about cryopreserved oocytes.

Attempts to directly determine the quality of oocytes using morphological evaluation have not yet determined reliable biomarkers to predict its reproductive potential. Instead of directly analyzing oocytes, reproductive medicine may better rely on indirect indicators analyzing data from large numbers of ART cycles. The overall potential for an oocyte to lead to a live birth is a metric, often called oocyte-to-baby rate. It can be determined from the number of oocytes collected and the cumulative live birth obtained through use of all those oocytes in subsequent embryo transfer cycles (fresh and frozen). Using cumulative data, different groups have attempted to calculate an oocyte-to-baby rate [3, 4]. Interestingly, two groups came up with a roughly similar calculation, namely about 20–23 oocytes are needed for the birth of one child, which corresponds to an oocyte potential of 5–4.3% for a childbirth. This value is relatively constant for women up to 37 years of age and, overall, largely depends on the ovarian response and the number of collected oocytes [4]. From the age of 38 years onwards, a live birth strongly depends on woman’s age and 43-year-old women would need more than 120 oocytes to reach maternity [4].

Although this data is derived from high number of cases in different clinical situations, the relatively low oocyte potential for a live birth may seem disappointing. This raises the question as to whether the low success rate reflects clinical or laboratory ART insufficiencies or if whether it is derived from an intrinsic “low” human biological reproductive efficiency.

To answer such questions, one approach could be to assess ART results alongside natural cycles data. Comparing to other species, including non-human primates, human reproductive efficiency seems relatively low. The probability of conception in one cycle is “only” about 20% [5]. It is also believed that during thousands of years, a major cause of death of healthy women in their reproductive age was related to pregnancy and labor. In such conditions, fewer offspring would instead increase the overall species survival [6]. Therefore, pondering evolutionary arguments, humans should not be considered to have “low,” but instead adequate reproductive efficiency. While accepting that male fertility is a factor, “low” fecundability per ovarian cycle can be considered from the perspective of the oocyte and its cellular and subcellular components. Therefore, the values for oocyte potential for a live birth must be regarded as adequate or fit, not “low,” and data from natural cycles should confidently be taken as reference values.

Nowadays, donated oocytes are often also cryopreserved before they are used in heterologous assisted reproduction. In retrospective studies estimating cumulative oocyte-to-baby rates in donors, representing best prognosis, in average, about 15 oocytes were found necessary to result in one live birth, corresponding to an oocyte potential of around 6.5% [3]. As expected, these values are more enthusiastic compared to the same age group of infertility patients (22–23 oocytes).

Although data from some donor MAR centers show comparable ART outcomes with fresh and cryopreserved donated oocytes [2], it is interesting that large data registries increasingly report significant differences in live birth rates between these two groups of donors [7]. The American Society for Reproductive Medicine (ASRM) guidelines also warn to be cautious about equating the development potential of fresh and cryopreserved oocytes and conclude that there is insufficient evidence that the live birth rate is the same with vitrified vs. fresh donor oocytes [8].

Today, vitrification is almost exclusively used, being slow freezing less common. There are many studies showing some effects of cryopreservation procedures, slow freezing and vitrification, on oocyte aneuploidy rate, sister chromatid exchange rate, DNA fragmentation, and methylation [9]. Cryopreservation procedures, freezing and vitrification, may also negatively affect the gene expression profile of human oocytes. Despite the removal of the label of experimental method about 10 years ago, detailed knowledge of these influences remains poor.

Regarding age, while women between 23 and 37 years old exhibit a fresh oocyte potential of 4.5% for a live birth, this figure drops has been estimated to decrease to 0.8% in a female at the age of 43 [4].

The critical question for patients and fertility specialists regarding the success with cryopreserved oocytes thus becomes: How many eggs need to be cryopreserved to achieve at least one child?

**Fig. 1:**
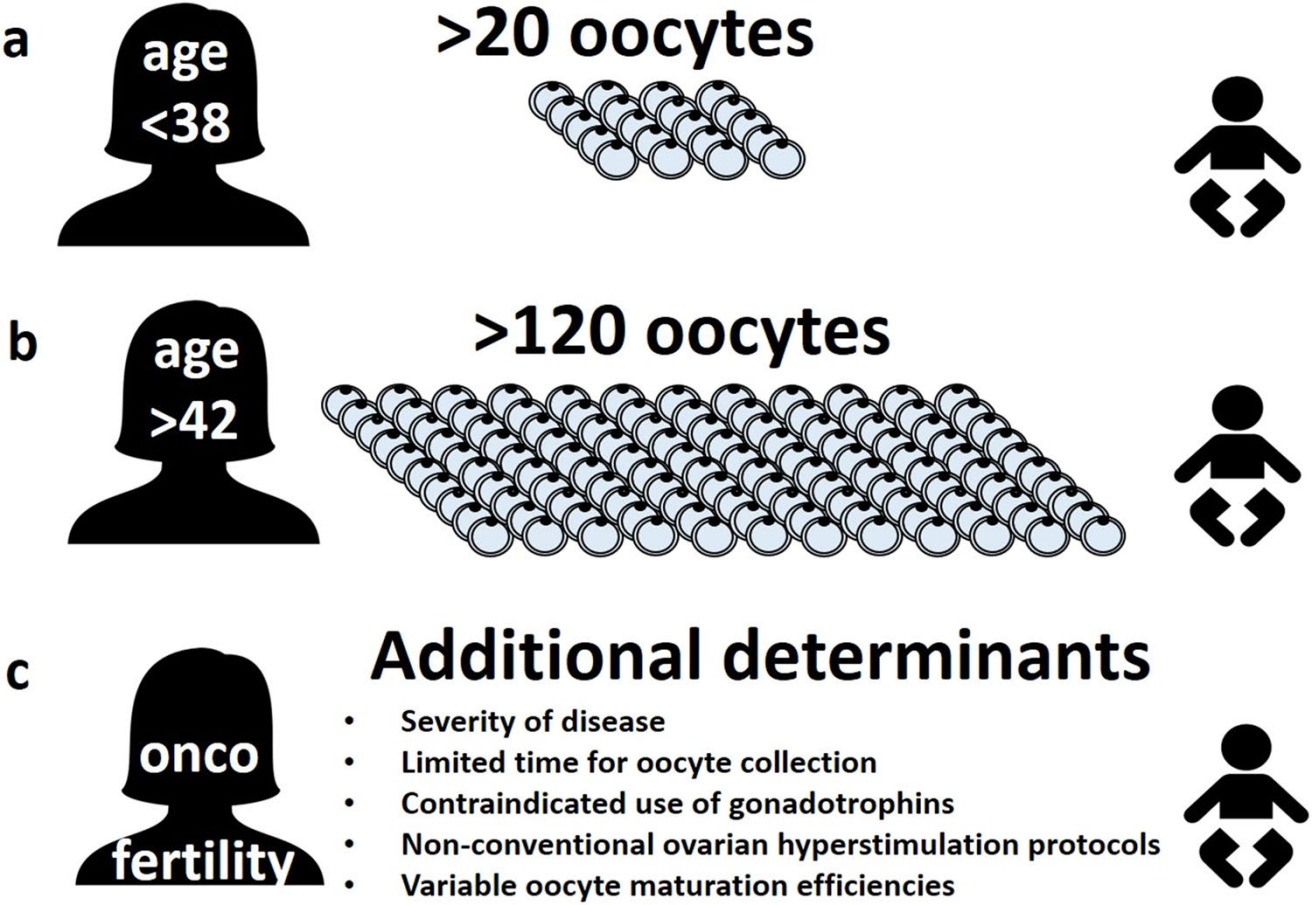
Information for patients about reproductive potential of cryopreserved oocytes to result in one live birth. **a**, **b** In planned fertility preservation, woman’s age and oocyte number are major determinants for a live birth; the estimation was calculated for around 80% likelihood of achieving at least one child. **c** In serious disease patients, there may be additional factors to consider influencing the probability for a live birth from cryopreserved oocytes; another problem in building a mathematical model for women with medical oocyte cryopreservation represents a low return rate (< 10%)

In a recent study on live birth outcomes after fertility preservation through oocyte vitrification for non-oncologic reasons, Goldman et al. [10] reported the clear effect of woman’s age and number of oocytes at time of collection. However, since a reduced number of women returned to use their cryopreserved oocytes in fertility preservation situations, models were put forward based on data recovered from a comparable subgroup of ART patients [10]. Extrapolating from male factor or tubal factor infertility situations, a model could be built to predict at least one live birth based on two variables: woman’s age and the number of mature oocytes obtained [10]. They estimated that more than 20 oocytes are needed to have around 80% chance of achieving at least one child at woman’s age below 38 (Fig. 1a) and more than 120 oocytes for the same chance at woman’s age above 42 years (Fig. 1b). This model may be too optimistic because besides extrapolating from retrospective data of a subgroup of ART patients, it also assumes high survival of warmed oocytes (95% for women below 36 years or 85% for woman above 35 years), and a 60% live birth rate per transferred blastocyst. The model also incorporates age-based euploidy rates into the predictions. So, it does not assume a 60% live birth rate for any transferred blastocyst, but instead estimates the likelihood that a given blastocyst would be euploid based on age, and then assumes the 60% live birth rate per transferred euploid blastocyst [10]. We propose predictive models of ART success after oocyte cryopreservation should be systematically used because they provide opportunities for more informative discussion between healthcare professionals and patients regarding expectations towards a live birth after one or more cycles of oocyte cryopreservation. This is important in particular for women of late reproductive age because the upper age limits for oocyte cryopreservation should not be uniformly set in legislation [1]. More difficult problem to dialogue with patients aiming for fertility preservation regards how to integrate realistic oocyte potential data with expectations for a live birth in different clinical situations. For example, a given number of collected oocytes may need to be considered as sufficient in some oncologic situations, while it may be discussed as insufficient in planned age-related situations. While this may be a difficult task to health workers leading with these patients, the use of such models allows additional opportunities for explanations (Fig. 1c). Due to low return rate (< 10%) of women with medical oocyte cryopreservation [11], the literature on oocyte-to-baby rate in these patients is clearly unbalanced with studies on oocyte donation cycles. This brings additional challenges in building accurate mathematical models predicting success in women with medical oocyte cryopreservation.

In patients with oncological diseases, the most important negative factor for live birth is the limitation of time to obtain enough oocytes or the contraindication for the use of gonadotropins. As a result, unconventional ovarian stimulation protocols may be used, which have not yet been confirmed to be comparable with classical protocols. Often, the only option remains cryopreservation after in vitro oocyte maturation, which further adds unevaluated factors to the fertility potential of such oocytes [12].

National and international ART registries should be improved by extending the set of variables about oocyte donation and oocyte cryopreservation cycles and better describing clinical situation characteristics, including woman’s age, both at time of collection and of embryo transfer, ovarian stimulation and endometrial preparation protocols, ovarian and uterine response, as well of specific existing pathologies. Such an expanded data set would allow for balance the literature about fertility potential of cryopreserved oocytes from young donors with the literature about oocyte fertility potential from women with various pathologic situations, resulting in more accurate and reliable predictive models in the future.
